# *The WHO AWaRe (Access, Watch, Reserve) antibiotic book* and prevention of antimicrobial resistance

**DOI:** 10.2471/BLT.22.288614

**Published:** 2023-02-10

**Authors:** Veronica Zanichelli, Michael Sharland, Bernadette Cappello, Lorenzo Moja, Haileyesus Getahun, Carmem Pessoa-Silva, Hatim Sati, Catharina van Weezenbeek, Hanan Balkhy, Mariângela Simão, Sumanth Gandra, Benedikt Huttner

**Affiliations:** a Department of Health Products Policy and Standards, World Health Organization, Avenue Appia 20, 1211, Geneva, Switzerland.; b Centre for Neonatal and Paediatric Infection, St George’s University of London, London, England.; c Department of Global Coordination and Partnership on Antimicrobial Resistance, World Health Organization, Geneva, Switzerland.; d Department of Surveillance, Prevention & Control of Antimicrobial Resistance, World Health Organization, Geneva, Switzerland.; e Antimicrobial Resistance Division, World Health Organization, Geneva, Switzerland.; f Access to Medicines and Health Products Division, World Health Organization, Geneva, Switzerland.; g Department of Internal Medicine, Division of Infectious Diseases, Washington University School of Medicine, Saint Louis, United States of America.

## Abstract

Guidance on the appropriate use of antibiotics for common infections is lacking in many settings. The World Health Organization (WHO) has recently released *The WHO AWaRe (Access, Watch, Reserve) antibiotic book* which complements the *WHO Model list of essential medicines* and *WHO Model list of essential medicines for children*. The book gives specific guidance on the empiric use of antibiotics in the model lists with a strong emphasis on the AWaRe framework, which is centred around the risk of antimicrobial resistance development associated with the use of different antibiotics. Recommendations in the book cover 34 common infections in primary and hospital care both for children and adults. The book also includes a section on the use of the last-resort Reserve antibiotics, whose use should be restricted to very selected cases when an infection is confirmed or suspected to be caused by multidrug-resistant pathogens. The book highlights the use of first-line Access antibiotics or no antibiotic care if this is the safest approach for the patient. Here we present the background behind the development of the AWaRe book and the evidence behind its recommendations. We also outline how the book could be used in different settings to help reach the WHO target of increasing the proportion of global consumption of Access antibiotics to at least 60% of total consumption. The guidance in the book will also more broadly contribute to improving universal health coverage.

## Résumé

De nombreuses régions manquent d'encadrement relatif à l'usage d'antibiotiques pour des infections courantes. L'Organisation mondiale de la Santé (OMS) a récemment publié le *Livre sur les antibiotiques AWaRe (Access, Watch, Reserve) de l'OMS,* qui complète la *Liste modèle des médicaments essentiels* ainsi que la *Liste modèle des médicaments essentiels destinés à l'enfant, *toutes deux établies par l'OMS. Ce livre fournit des indications spécifiques concernant l'usage empirique des antibiotiques dans les listes modèles en insistant sur le cadre AWaRe, qui se concentre sur le risque d'apparition d'une résistance aux antimicrobiens en lien avec le recours à différents antibiotiques. Les recommandations du livre portent sur 34 infections courantes dans les soins primaires et hospitaliers, tant chez les adultes que chez les enfants. Cet ouvrage inclut en outre une section dédiée à l'utilisation en dernier recours des antibiotiques appartenant à la catégorie Reserve, à employer uniquement dans des cas très précis où des pathogènes multirésistants constituent la cause suspectée ou avérée de l'infection. Il privilégie l'emploi d'antibiotiques de première intention appartenant à la catégorie Access, voire l'absence de recours aux antibiotiques s'il s'agit de l'approche la plus sûre pour le patient. Dans le présent document, nous définissons le contexte dans lequel le livre AWaRe a été rédigé, ainsi que les preuves à l'origine des recommandations formulées. Nous exposons également dans les grandes lignes la manière dont ce livre peut être utilisé à divers endroits pour contribuer à la réalisation de l'objectif fixé par l'OMS: accroître le pourcentage global de consommation des antibiotiques de la catégorie Access afin qu'il représente au moins 60% de la consommation totale. Enfin, les conseils prodigués par l'ouvrage aideront plus généralement à améliorer la couverture sanitaire universelle.

## Resumen

Muchas poblaciones carecen de orientaciones sobre el uso adecuado de antibióticos para tratar las infecciones más comunes. La Organización Mundial de la Salud (OMS) ha publicado recientemente *El libro de antibióticos AWaRe (Access, Watch, Reserve) de la OMS*, que complementa la *Lista modelo de medicamentos esenciales de la OMS* y la *Lista modelo de medicamentos pediátricos esenciales de la OMS*. El libro ofrece orientaciones específicas sobre el uso empírico de antibióticos en las listas modelo, destacando el marco AWaRe, que se centra en el riesgo de desarrollo de resistencia a los antimicrobianos asociada al uso de diferentes antibióticos. Las recomendaciones del libro abordan 34 infecciones comunes en atención primaria y hospitalaria, tanto en niños como en adultos. El libro también incluye una sección sobre el uso de los antibióticos en la categoría Reserve (reserva) de último recurso, cuyo uso se debe restringir a casos muy seleccionados cuando se confirma o se sospecha que una infección está causada por patógenos multirresistentes. El libro destaca el uso de antibióticos en la categoría Access (acceso) de primera línea o la no administración de antibióticos si este es el enfoque más seguro para el paciente. En el presente documento, exponemos el contexto en el que se ha desarrollado el libro AWaRe y las evidencias en las que se basan sus recomendaciones. También describimos cómo se podría utilizar el libro en diferentes contextos para ayudar a alcanzar el objetivo de la OMS de aumentar el porcentaje de consumo global de antibióticos en la categoría Access hasta al menos el 60 % del consumo total. Por último, las orientaciones incluidas en el libro contribuirán a mejorar la cobertura sanitaria universal de manera más general.

## ملخص

تفتقر العديد من الأماكن للتوجيهات الخاصة بالاستخدام المناسب للمضادات الحيوية في حالات العدوى الشائعة. أصدرت منظمة الصحة العالمية (WHO) مؤخرًا كتاب منظمة الصحة العالمية * AWaRe *حول المضادات الحيوية * ( *الوصول والمراقبة الاحتياطي *) * الذي يتكامل مع القائمة النموذجية للأدوية الأساسية التابعة لمنظمة الصحة العالمية والقائمة النموذجية للأدوية الأساسية للأطفال التابعة لمنظمة الصحة العالمية. يقدم الكتاب توجيهات محددة حول الاستخدام التجريبي للمضادات الحيوية في قوائم النماذج، مع تركيز قوي على إطار عمل AWaRe، والذي يتمحور حول مخاطر تطوير مقاومة مضادات الميكروبات المرتبطة باستخدام المضادات الحيوية المختلفة. تغطي التوصيات الواردة في الكتاب 34 إصابة شائعة في الرعاية الأولية، ورعاية المستشفيات لكل من الأطفال والكبار على حد سواء. يتضمن الكتاب أيضًا قسمًا عن استخدام المضادات الحيوية الاحتياطية التي تعد الملاذ الأخير، ويجب أن يقتصر استخدام هذه المضادات الحيوية على حالات محددة جدًا، عندما يتم تأكيد الإصابة، أو الاشتباه في أنها ناجمة عن مسببات الأمراض المقاومة للأدوية المتعددة. يسلط الكتاب الضوء على استخدام المضادات الحيوية للوصول من الدرجة الأولى، أو الرعاية دون المضادات الحيوية إذا كان هذا هو النهج الأكثر أمانًا للمريض. نحن نقدم هنا الخلفية وراء تطوير كتاب AWaRe، والأدلة وراء توصياته. نحن أيضًا نحدد كيف يمكن استخدام الكتاب في أماكن مختلفة للمساعدة في الوصول إلى هدف منظمة الصحة العالمية، المتمثل في زيادة نسبة الاستهلاك العالمي من المضادات الحيوية للوصول إلى %60 على الأقل من إجمالي الاستهلاك. كما سوف تسهم توجيهات الكتاب على نطاق أوسع في تحسين التغطية الصحية الشاملة.

## 摘要

在许多情况下，在适当使用常见感染用抗生素药物方面均缺乏指导。世界卫生组织 (WHO) 近期发布了*世界卫生组织 AWaRe 【可广泛使用 (Access)、谨慎使用 (Watch) 和保留使用 (Reserve)】抗生素药物目录*，这是对*世界卫生组织基本药物标准清单*和*世界卫生组织儿童基本药物标准清单*的补充。该目录为标准清单中抗生素药物的基于经验的使用提供了具体指导，突出强调 AWaRe 框架。该框架重点介绍由使用多种抗生素产生的抗菌素耐药性所带来的风险。目录中的建议涵盖了儿童和成年人初级和医院护理中的 34 种常见感染情况。同时，目录还包括了关于终极手段保留使用 (Reserve) 抗生素药物的使用说明部分，此类用途应仅限于当确诊或疑似为由多药耐药病原体引发感染的少数特定病例。目录重点介绍了一线可广泛使用 (Access) 抗生素药物的使用以及不使用抗生素的护理（如果这是患者最安全的治疗方法）。我们特此介绍了编制 AWaRe 目录的背景以及目录中建议背后的循证信息。同时我们还概述了在不同环境中如何使用该目录，以帮助实现世界卫生组织将全球范围内可广泛使用 (Access) 抗生素药物的使用量提升至至少占抗生素药物总使用量 60% 的目标。目录中提供的指导也将更广泛地促进全民健康覆盖率的提高。

## Резюме

Во многих учреждениях отсутствуют руководства по надлежащему применению антибиотиков при распространенных инфекциях. Всемирная организация здравоохранения (ВОЗ) недавно выпустила справочник по антибиотикам *The WHO AWaRe (Access, Watch, Reserve) antibiotic book*, который дополняет *изданный ВОЗ* *Примерный перечень основных лекарственных средств* и *изданный  ВОЗ* *Примерный перечень основных лекарственных средств для детей*. В справочнике даны конкретные рекомендации по эмпирическому использованию антибиотиков в примерных перечнях с особым упором на классификацию AWaRe, посвященную риску развития устойчивости к антимикробным препаратам вследствие использования различных антибиотиков. Приведенные в справочнике рекомендации охватывают 34 распространенные инфекции, которые встречаются при оказании первичной медико-санитарной помощи и при лечении в стационаре как у взрослых, так и у детей. Справочник также включает раздел о резервных антибиотиках, предназначенных для применения в крайнем случае. Их использование должно ограничиваться очень избранными случаями, когда подтверждено или подозревается, что инфекция вызвана мультирезистентными патогенами. В справочнике подчеркивается необходимость использования антибиотиков первой линии группы доступа или отсутствия лечения антибиотиками, если это наиболее безопасный подход к здоровью пациента. В данном материале представлена история создания справочника AWaRe и доказательная база, лежащая в основе его рекомендаций. Также приводится описание возможных способов использования справочника в различных условиях для содействия достижению цели ВОЗ по увеличению доли мирового потребления антибиотиков группы доступа (Access) как минимум до 60% от общего потребления. Приведенные в справочнике рекомендации также будут способствовать улучшению всеобщего охвата услугами здравоохранения в более широком смысле.

## Introduction

Optimizing the use of antibiotics is a key objective of the WHO *Global action plan on antimicrobial resistance*.^1^ However, the lack of comprehensive evidence-based guidance for common infections is a major barrier for optimal use in many settings.^2^

In 2001, WHO published the *WHO Model prescribing information: drugs used in bacterial infections*,^3^ which provided advice to health professionals, especially in Sub-Saharan Africa. WHO has now developed a new book, the *WHO AWaRe (Access, Watch, Reserve) antibiotic book,*^4^ with the aim of providing simple guidance for the optimal empiric treatment of common bacterial infections in children and adults. This new book targets health-care providers in all types of income settings as antimicrobial resistance is a global problem. The book is closely aligned to the *WHO Model list of essential medicines*, *WHO Model list of essential medicines for children* and WHO’s AWaRe classification of antibiotics.^5^^–^^7^ WHO created the AWaRe classification in 2017 (and revised it in 2019 and 2021) to categorize antibiotics into three groups: Access, Watch and Reserve (Box 1). The book builds on the AWaRe classification and model lists for both children and adults by optimizing the quantity and quality of antibiotic prescribing, and focuses on improving the use of narrow-spectrum Access antibiotics, reducing the overuse of oral Watch antibiotics, and where appropriate encourages symptomatic care with no antibiotic treatment.^4^^,^^8^ WHO has set a target that at least 60% of overall country-level antibiotic use should be from the Access group by 2023, to contain rising resistance and make antibiotic use safer and more effective.^9^

Box 1 **Access, Watch and Reserve antibiotics definitions**

- **Access** antibiotics are antibiotics with a narrow spectrum of activity, generally with less side-effects, a lower potential for the selection of antimicrobial resistance and of lower cost. They are recommended for the empiric treatment of most common infections and should be widely available.
- **Watch **antibiotics generally have a higher potential for the selection of antimicrobial resistance and are more commonly used in sicker patients in the hospital facility setting. Their use should be carefully monitored to avoid overuse.
- **Reserve **antibiotics are last-resort antibiotics that should only be used to treat severe infections caused by multidrug-resistant pathogens.

## AWaRe

WHO and others are increasingly employing the AWaRe classification to monitor antibiotic use and support antimicrobial stewardship activities.^10^^,^^11^ In the 2021 update of AWaRe classification, 257 antibiotics used worldwide have been classified into AWaRe groups (including all 39 antibiotics listed in the 2021 *WHO Model list of essential medicines* and 36 antibiotics in the *WHO Model list of essential medicines for children*).^12^^–^^16^ The 2021 model lists include 31 Access and Watch antibiotics (about 6% of the 479 medicines inthe model lists).^5^^,^^6^ These antibiotics cover first or second choice options for the empiric treatment of 34 common infections in the primary health care and hospital facility settings. In addition, eight Reserve antibiotics are listed for the treatment of multidrug-resistant pathogens (Box 2).^5^

Box 2 **Access, Watch and Reserve antibiotics in the 2021 *WHO Model list of essential medicines* and *WHO Model list of essential medicines for children***

**Access group**

Amikacin; amoxicillin; amoxicillin + clavulanic acid; ampicillin; benzathine benzylpenicillin; benzylpenicillin; cefalexin; cefazolin; chloramphenicol; clindamycin; cloxacillin; doxycycline; gentamicin; metronidazole; nitrofurantoin; phenoxymethylpenicillin; procaine benzylpenicillin; spectinomycin (not listed in the model list for children); sulfamethoxazole + trimethoprim; and trimethoprim.

**Watch group**

Azithromycin; cefixime; cefotaxime; ceftazidime; ceftriaxone; cefuroxime; ciprofloxacin; clarithromycin; meropenem; piperacillin + tazobactam; and vancomycin.

**Reserve group**

Cefiderocol (not listed in the model list for children); ceftazidime + avibactam; colistin; fosfomycin; linezolid; meropenem + vaborbactam (not listed in the model list for children); plazomicin (not listed in the model list for children); and polymyxin B.

In a meta-analysis, researchers assessed the risk of colonization or infection by multidrug-resistant pathogens after exposure to antibiotics classified by the AWaRe categories.^10^ While there are limitations in the primary studies included in the meta-analysis, the results clearly show that exposure to almost all antibiotics was associated with an increased risk of colonization or infection with any multidrug-resistant organism. The use of Watch antibiotics was associated with about twice the risk of subsequent colonization or infection with a multidrug-resistant pathogen compared to not having been exposed to Watch antibiotics. For example, 3rd generation cephalosporins exposure increased the risk for acquiring Enterobacterales producing extended-spectrum β-lactamases (odds ratio, OR: 2.5; 95% confidence interval, CI: 2.2–2.9); quinolones exposure increased the risk for methicillin-resistant *Staphylococcus aureus* (OR: 2.2; 95% CI: 1.8–2.7); glycopeptides exposure increased the risk for vancomycin-resistant *Enterococcus* (OR: 2.7; 95% CI: 2.2–3.2); and carbapenems exposure increased the risk for carbapenem-resistant *Acinetobacter baumannii* (OR: 2.2; 95% CI: 1.8–2.6), carbapenem-resistant Enterobacterales (OR: 2.5; 95% CI: 2.2–2.7) and carbapenem-resistant *Pseudomonas aeruginosa* (OR: 3.2; 95% CI: 2.5–4.2). These findings support the principles of *The WHO AWaRe (Access, Watch, Reserve) antibiotic book*, which is to increase the proportion of patients treated with Access antibiotics or no antibiotic treatment where appropriate.

## Development of the book

The book was developed by the Essential Medicines List Secretariat with the support of the Essential Medicines List Antimicrobial Working Group. The book builds around the recommendations of using specific antibiotics for specific infections given in the 2017 model lists. The development of these recommendations for specific antibiotics for the treatment of common acute infections followed an evidence-based and open process that included assessments of more than 1000 clinical trials, systematic reviews and international clinical practice guidelines (Box 3). To ensure transparency, inclusiveness and adequacy of the information provided in the book, a public consultation on the draft of the book was held between November 2021 and January 2022. Based on the input received, the authors extensively revised some sections.^17^ The book is not a formal WHO guideline, although the choice of which antibiotics to use for a specific infection represent formal recommendations. The book also provides high quality medical information for the diagnosis, symptomatic care, dosing and treatment duration for specific infections. The information contained in the book is based on valid, up-to-date literature and expert consensus within the WHO Essential Medicines List Antimicrobial Working Group. To inform decisions when empiric choice may need to differ from a recommendation due to local resistance patterns, the authors used microbiology data from the WHO Global Antimicrobial Resistance and Use Surveillance System whenever possible. Since 2016, the surveillance system reports country-level resistance data for pathogens that commonly cause acute human infections using samples collected routinely by more than 100 countries enrolled in the system.^16^^,^^18^ For example, using the surveillance data about resistance in *Salmonella* spp. blood culture isolates,^18^ the authors revised the empiric treatment of enteric fever by giving two different options based on local risk of fluoroquinolone resistance (Table 1). Strengthening the generation of representative quality microbiology data that is ideally linked with clinical information would be key to inform the local implementation of the book. Furthermore, the book is both building on and closely connected to other WHO antimicrobial resistance initiatives including the stewardship toolkit,^19^ the overview and analysis of antimicrobials in clinical and preclinical development antibiotics,^20^ WHO’s list of critically important antimicrobials for human medicine,^21^ WHO’s list of priority pathogens^22^ and the third WHO Model list of essential in vitro diagnostics.^23^

Box 3 **Process of the *WHO Model list of essential medicines* and *WHO Model list of essential medicines for children* updates**

2015:

- Global action plan on antimicrobial resistance adopted by the World Health Assembly

2016:

- The Essential Medicine List Secretariat established an Essential Medicine List Antimicrobial Working Group composed of global experts to update the list of antibiotics in the *WHO Model list of essential medicines* and *WHO Model list of essential medicines for children*
- The working group evaluated the evidence from systematic reviews and meta-analyses of randomized controlled trials of antibiotic treatment for all relevant infections and international clinical practice guidelines
- The working group delegated the task of reviewing the evidence to the Centre for Infectious Diseases, Research Methods and Recommendations of McMaster University, Hamilton, Canada
- The Essential Medicine List Expert Committee reviewed the antibiotics proposed by the working group and made the final recommendations on antibiotics for inclusion in both model lists

2017:

- AWaRe framework introduced (initially only for antibiotics on the Essential Medicines List)
- Update of both model lists: a major revision of antibiotics in the list is conducted following the comprehensive evaluation of systematic reviews and meta-analyses of randomized controlled trials and clinical practice guidelines for the treatment of common infections

2019:

- AWaRe revised (expanded to all antibiotics). Introduction of a “not recommended” category
- Update of both model lists: antibiotic treatment for two additional infections (typhoid fever and oral or dental infections) and surgical prophylaxis were reviewed

2021:

- AWaRe revised
- Update of both model lists: antibiotic treatment for two additional infections was reviewed (eye infections, bronchitis)

**Table 1:** Example of how surveillance data can inform the need for different initial empiric treatments based on local resistance thresholds

Antibiotic^a^	% of patients with resistant *Salmonella* spp bloodstream infections, median (IQR)	Recommendation in the WHO AWaRe Book
Ciprofloxacin	20 (5–35)^b^	1^st^ choice if low risk of fluoroquinolone resistance
Ceftriaxone	5 (0–15)^c^	1^st^ choice if high risk of fluoroquinolone resistance

IQR: interquartile range; WHO: World Health Organization.

^a^ Only antibiotics recommended in the *WHO Model list of essential medicines* and *WHO Model list of essential medicines for children* for the treatment of enteric fever are reported. Azithromycin is also recommended in both model lists for the treatment of enteric fever if the risk of fluoroquinolone resistance is high, but no surveillance data from WHO Global Antimicrobial Resistance and Use Surveillance System for this pathogen and antibiotic combination is currently available.

^b^ Data reported to the WHO Global Antimicrobial Resistance and Use Surveillance System in 2020 from 32 countries and 11 483 infections.

^c^ Data reported to the WHO Global Antimicrobial Resistance and Use Surveillance System in 2020 from 26 countries and 11 009 infections.

Data source: WHO (2021).^18^

## Content of the book

The book includes individual chapters for 34 common clinical infections in children and adults in both the primary health care and hospital settings. Each chapter provides information regarding the clinical management of the infection, common clinical presentation (of mild and severe cases) and key diagnostic tests to consider. Each chapter is focused on antibiotic treatment strategies at the time of first clinical presentation (that is, empiric treatment rather than targeted treatment based on microbiology results and an antibiogram), when a working clinical diagnosis has been made based on the patient’s history and examination, but test results are not yet available (Box 4 and Box 5).

Box 4 **First choice antibiotic options indicated in the WHO AWaRe Book for the treatment of common infections in primary health care**

- **Bronchitis**: no antibiotic
- **Acute otitis media**: amoxicillin (A)
- **Pharyngitis**: phenoxymethylpenicillin (A) or amoxicillin (A)
- **Acute sinusitis**: amoxicillin (A) or amoxicillin + clavulanic acid (A)
- **Dental infections**: amoxicillin (A) or phenoxymethylpenicillin (A)
- **Acute localized lymphadenitis**: amoxicillin + clavulanic acid (A) or cefalexin (A) or cloxacillin (A)
- **Mild community-acquired pneumonia**: amoxicillin (A) or phenoxymethylpenicillin (A)
- **COPD exacerbations**: amoxicillin (A)
- **Infectious bloody diarrhoea or dysentery**: ciprofloxacin (W)
- **Enteric fever**: ciprofloxacin (W) or azithromycin or ceftriaxone (W)
- **Skin and soft tissue infections (impetigo, erysipelas, cellulitis)**: amoxicillin + clavulanic acid (A) or cefalexin (A) or cloxacillin (A)
- **Burn and wound-related infections**: amoxicillin + clavulanic acid (A) or cefalexin (A) or cloxacillin (A)
- **Lower urinary tract infections**: nitrofurantoin (A) or sulfamethoxazole + trimethoprim (A) or trimethoprim (A) or amoxicillin + clavulanic acid (A)

COPD: chronic obstructive pulmonary disease.Note: (A) denotes Access antibiotics and (W) denotes Watch antibiotics.

Box 5 **First choice antibiotic options indicated in the WHO AWaRe Book for the treatment of infections commonly encountered in the hospital setting**

- **Sepsis (adults):** ceftriaxone (W) or cefotaxime (W) combined with gentamicin (A) or amikacin (A)
- **Sepsis (neonates or children):** ampicillin (A) or benzylpenicillin (A) combined with gentamicin (A)
- **Bacterial meningitis (adults or children**): ceftriaxone (W) or cefotaxime (W)
- **Bacterial meningitis (neonates):** ampicillin (A) combined with gentamicin (A)
- **Severe community-acquired pneumonia:** ceftriaxone (A) or cefotaxime (A) combined with clarithromycin (A)
- **Community-acquired pneumonia (children):** ampicillin (A) or amoxicillin (A) or benzylpenicillin (A) combined with gentamicin (A)
- **Hospital-acquired pneumonia:** amoxicillin + clavulanic acid (A) or ceftriaxone (W) or cefotaxime (W) or piperacillin + tazobactam (W)
- **Mild intra-abdominal infection**^a^: amoxicillin + clavulanic acid (A) or ceftriaxone (W) or cefotaxime (W) combined with metronidazole (A)
- **Severe intra-abdominal infection**^a^: piperacillin + tazobactam (W) or ceftriaxone (W) or cefotaxime (W) combined with metronidazole (A)
- **Mild upper urinary tract infection:** ciprofloxacin (W)
- **Severe upper urinary tract infection:** ceftriaxone (W) or cefotaxime (W) combined with gentamicin (A) or amikacin (A)
- **Bone and joint infection**^b^: cloxacillin (A)
- **Severe skin and soft tissue infections (necrotizing fasciitis**)**:** piperacillin + tazobactam (W) combined with clindamycin (A) or ceftriaxone (W) combined with metronidazole (A)
- **Severe skin and soft tissue infections (pyomyositis):** amoxicillin + clavulanic acid (A) or cefalexin (A) or cloxacillin (A)
- **Febrile neutropenia (with low risk of serious infection):** amoxicillin + clavulanic acid (A) combined with ciprofloxacin (W)
- **Febrile neutropenia (with high risk of serious infection):** piperacillin + tazobactam (W)
- **Surgical prophylaxis**^c^: cefazolin (A) or cefazolin (A) combined with metronidazole (A)

^a^ Appendicitis, cholangitis/cholecystitis, diverticulitis, pyogenic liver abscess.^b^ Septic arthritis and osteomyelitis.^c^ The choice of prophylaxis depends on the type of surgical procedure.Note: (A) denotes Access antibiotics and (W) denotes Watch antibiotics.

Clinical signs and symptoms to assess the risk of more complicated courses (e.g. based on the clinical presentation, underlying disease and the presence of risk factors for infections caused by resistant bacteria) are summarized in each infection chapter.

All chapters are complemented by infographics summarizing the key information for each infection in a user-friendly, quickly accessible format. A mobile application of the book contents, and the book in e-pub format are also freely available and can be used both on- and offline.^24^

### Symptomatic care

A key aim of the book is to highlight low-risk cases of certain infections where the preferred approach is to observe and wait, providing symptomatic treatment but no antibiotics, based on the initial clinical risk assessment for severity and complications. For example, mild infections, often likely to be of viral origin, in otherwise healthy individuals, could be treated in such a way to avoid the unnecessary use of antibiotics. Box 6 shows examples of instances when this approach can be used. Each infection chapter is providing elements to help identify these cases.

Box 6 **Infections in which no antibiotic care may be the appropriate first line treatment**

***Acute diarrhoea***

Most cases do not require antibiotic treatment^a^ because the infection is of viral origin and the illness is usually self-limiting regardless of the causative pathogen. The cornerstone of treatment is rehydration.

***Bronchitis***

Nearly all cases have a viral origin and there is no evidence that antibiotics are needed.

***Mild COPD exacerbations***

Most exacerbations are not triggered by bacterial infections; only certain cases will benefit from antibiotic treatment.

***Dental infections***

Dental treatment rather than prescribing antibiotics is generally more appropriate in the management of dental infections.

***Otitis media***

Most non-severe cases can be managed symptomatically and do not require antibiotic treatment.

***Pharyngitis***

Most cases do not require antibiotics because the infection is commonly viral.

***Sinusitis***

Most cases do not require antibiotics because the infection is commonly viral.

***Mild skin and soft tissue infections***

In cases of wounds at low risk of becoming infected, antibiotic treatment is not needed.

In cases of animal bites, only wounds in high-risk anatomical locations and patients with severe immunosuppression benefit from antibiotic treatment.

***Lower urinary tract infections^b^***

In young women who are not pregnant, with mild symptoms and who may wish to avoid or delay antibiotic treatment, symptomatic treatment alone can be considered.

COPD: chronic obstructive pulmonary disease.^a^ Unless significant bloody diarrhoea is present.^b^ Symptomatic care as first line treatment for lower urinary tract infections only applies to a few selected patients with no risk factors for complicated infections.

### Dosing and duration

The book gives appropriate information on dosing and treatment duration with an emphasis on encouraging treatment for the shortest acceptable duration. An early switch from intravenous to oral treatment is strongly encouraged to enable clinically appropriate hospital discharge as soon as possible. The section on dose and duration is aligned with guidance developed by the United Kingdom National Institute for Health and Care Excellence, which is based on extensive evidence-based reviews of the literature.^25^

### Reserve antibiotics

Reserve antibiotics are selected for the *WHO Model list of essential medicines* based on their public health utility. For each of these last-resort antibiotics, guidance is provided on where their targeted and empiric use might be considered appropriate, along with practical prescribing information. The guidance fully acknowledges that local factors, such as availability of microbiology laboratories, need to be considered, and avoidance of inappropriate use of these antibiotics must be a priority.

## Way forward

While updating the WHO guidance on optimal antibiotic use for common infections took 20 years, it is important that antibiotic recommendations in the book are updated regularly. Updating a single globally relevant guidance that covers dozens of diseases and multiple treatment options is challenging. The use of the WHO Global Antimicrobial Resistance and Use Surveillance System, along with closer linkages between people updating the model lists and other WHO antimicrobial resistance stewardship programmes and policy initiatives will facilitate the process. 

While comprehensive antibiotic guidance is a crucial component of any antimicrobial resistance stewardship programme, it has a limited impact as an isolated intervention.^26^ For future guidance, improving the evidence base is needed, particularly addressing the lack of clinical trials in low- and middle-income countries focusing on antibiotic prescribing strategies in common infections. Of the 1070 clinical trials from 1954 and onwards that informed the update of the 2017 model lists, approximately 300 (28%) trials had been performed in low- and middle-income countries, of which less than 10 trials (< 1%) were conducted in low-income countries.^27^ The paucity of representative data from low- and middle-income countries is an important limitation for the generalization of empiric guidance. Improved local data and risk assessments are needed to adapt recommendations at local level. Worldwide, there is a lack of community antimicrobial resistance and antimicrobial consumption surveillance data. Over 90% of antibiotic prescribing for human use occurs in primary care;^16^ however, the evidence from this setting to inform the optimal design of educational and stewardship interventions is insufficient. During the development of the book many research questions have emerged, particularly regarding optimal antibiotic treatment in low- and middle-income countries, such as: (i) How can clinical and microbiology surveillance data most effectively be combined and integrated into future country or local infection-specific adaptation of WHO guidance? (ii) What are appropriate thresholds of resistance in key pathogens that justify the empiric use of broader spectrum agents with wider coverage for resistant pathogens, both in community- and hospital-acquired infections? (iii) What type of microbiologic and clinical data is needed to define these thresholds? (iv) How feasible and appropriate is a risk-based prescribing approach for global guidance, and how can appropriate clinical risk scores be developed for low- and middle-income countries? (v) How should the choice of antibiotic, dosing and duration of treatment vary in severe infections, or for vulnerable populations and patients with significant underlying disease? (vi) What are the relative risks and benefits of symptomatic care and alternative antibiotic prescribing strategies for mild infections in primary care in the low- and middle-income country setting? and (vii) How does the guidance provided help to inform the development of appropriate and relevant country and global level policy metrics for levels of optimal prescribing using the AWaRe system?

From simply listing antibiotics in the adult and children model lists to providing detailed guidance on their optimal use in the book is a major step in ensuring equity of access. Policy-makers can use the information provided in the book to set policies that help in achieving several targets. Target 3.8 of the sustainable development goals identifies access to safe, effective, quality and affordable medicines for all as a key component of achieving universal health coverage (UHC). The clear and specific guidance in the book on the appropriate use of narrow- and broad-spectrum antibiotics listed in WHO’s model lists is an important step towards achieving UHC while at the same time preserving the antibiotics’ continued efficacy. By using the book, policy-makers can determine equity of access to effective antibiotics for specific clinical conditions in both the primary care and hospital setting. They will be able to determine not only if the medicine is available generally in the health-care facility, but also if it is available to treat a patient with a specific infection, at the right dose and duration, in a safe, effective, quality-assured and affordable formulation. With the target of at least 60% Access antibiotics in place, the book will support achieving the target by clarifying how detailed, timely, reliable and actionable data could now be derived on access to the essential AWaRe antibiotics. Using this approach to treat common infections would be an important step towards combatting antimicrobial resistance and creating healthier populations. 
